# Association of Major Chronic Noncommunicable Diseases and Life Expectancy in China, 2019

**DOI:** 10.3390/healthcare10020296

**Published:** 2022-02-03

**Authors:** Liang Sun, Yabing Zhou, Mengge Zhang, Chuancang Li, Mengbing Qu, Qian Cai, Jingjing Meng, Haohao Fan, Yang Zhao, Dongsheng Hu

**Affiliations:** 1 Department of Social Medicine and Health Management, College of Public Health, Zhengzhou University, Zhengzhou 450001, China; zzusunl@163.com (L.S.); zhouyabing@gs.zzu.edu.cn (Y.Z.); menggeyaya@163.com (M.Z.); lichuancang@yeah.net (C.L.); Qumengbing@163.com (M.Q.); 15638577525@163.com (Q.C.); mengjingjingen@163.com (J.M.); fanhaooa@163.com (H.F.); 2 Department of Epidemiology and Biostatistics, College of Public Health, Zhengzhou University, Zhengzhou 450001, China

**Keywords:** chronic non-communicable diseases, life table, cause-eliminated life expectancy

## Abstract

This study aimed to illustrate the association of four major chronic noncommunicable diseases (cardiovascular diseases, cancer, respiratory diseases, and diabetes) with life expectancy (LE) of Chinese residents in 2019 and to provide an evidence base for the scientific prevention and treatment of chronic diseases in China. The abbreviated life and cause-eliminated life tables were compiled according to the Jiang Qing Lang method recommended by WHO (World Health Organization) to calculate LE and cause-eliminated life expectancy (CELE) in 2019. The disease that had the greatest association with the LE of Chinese residents was cardiovascular disease (CVD), with the LE increasing by 8.13 years after removing CVD deaths. This was followed by cancer (2.68 years), respiratory diseases (0.88 years), and diabetes (0.24 years). The four major chronic noncommunicable diseases (NCDs) were the main diseases affecting the health of Chinese residents. CVD should be prevented and treated as the key disease among the chronic diseases, while women and rural people should be the major focus of health knowledge promotion. All residents should be encouraged to develop a good understanding of self-protection and of how to achieve a healthy lifestyle in order to reduce the occurrence of death and to improve their quality of life and health in general.

## 1. Introduction

In 1990, the United Nations Development Statistics Agency, in their Human Development Report [1], began using life expectancy (LE) as one of the indicators to be used for the measurement of the level of human development in individual countries. LE is a statistical measure of the average time an organism is expected to live based on current mortality [2]. It is a comprehensive indicator, reflecting the national socio-economic level, education level, health status of the population, and healthcare status. LE level is affected by many factors, but diseases and lifestyles are the direct factors that influence it [3]. Since the founding ceremony of the People’s Republic of China, with the development of a social economy and changes in people’s way of life, infant mortality rates and infectious disease mortality rates of Chinese residents have been gradually falling. The LE of Chinese people increased from 35 years before liberation to 77 years in 2019. With the increasing age of the population, the major burden of disease has shifted to chronic non-communicable diseases (NCDs), with deaths caused by chronic diseases becoming a major public health problem affecting not just the health of residents but the economic and social development of the country [4,5]. By evaluating the association of four chronic diseases (cardiovascular diseases, cancer, respiratory diseases, and diabetes) with the LE of Chinese residents, this paper provides the basis for improving the health level of residents and formulating corresponding prevention and control strategies, thus creating an environment conducive to improving LE.

By 2030 [6], China’s aging population will be responsible for an increase in the burden of chronic disease by at least 40 percent. According to the World Health Statistics Report 2020 [7], an estimated 41 million people, or 71 percent of all deaths, were from NCDs in 2016, with the leading causes being cardiovascular disease (CVD), cancer, chronic respiratory disease, and diabetes.

Currently, the study of LE in China mainly occurs at the provincial level, with a focus on chronic diseases such as CVD [8]. Due to differences in the economies, environment, medical insurance level, and lifestyles among different regions in China [9], the association between CVD, cancer, chronic respiratory disease, diabetes, and LE in China was studied in relation to people’s geographical situation [10,11]. Through in-depth analysis of the association of different disease types and different sexes (male and female), different areas (urban and rural), and different regions (eastern, central, and western) in 2019, high-risk groups were screened to provide reference points for formulating targeted disease prevention measures in China.

## 2. Material and Methods

### 2.1. Data Source

The data were collected from the China-cause of-death surveillance dataset of 2019, compiled by the National Health Commission Statistics Information Center and the China Center for Disease Control and Prevention Chronic Non-Communicable Diseases Prevention and Control Center (http://en.nhc.gov.cn/) (accessed on 15 December 2021). In 1989, a National Disease Surveillance System, or Death Surveillance Points System (DSPs), was formally established. The national cause of death surveillance system was formed by selecting representative disease surveillance sites in 31 provinces (autonomous regions and municipalities directly under the central government) through the principle of multi-stage stratified whole-group random sampling, forming a national disease surveillance system consisting of 605 disease surveillance sites, in which birth, population, and death information was routinely collected, enabling the monitoring of over 300 million people, covering 24% of the national population, with good provincial representation.

### 2.2. Registration of Deaths and Major Diseases of Death Coding

All deaths in the national cause-of-death surveillance system were reported online through the cause-of-death registration and reporting information system of the Chinese Center for Disease Control and Prevention, which reviews the data reported by the provinces, verifies it, and corrects any problems found.

Causes of death were coded according to the International Classification of Diseases, the 10th edition (ICD-10). The four major chronic diseases included CVD (I00-I99), cancer (C00-C97), respiratory diseases (J30-J98), and diabetes (E10-E14).

This study divided the population into five age groups, namely 0–4, 5–9, 10–14, and so on. Such a grouping can eliminate differences in population size by age, and it can better reveal the characteristics of the population as a whole.

### 2.3. Calculation of Life Expectancy and Cause-Eliminated Life Expectancy

The abbreviated life table was prepared using the method of Jiang Qinglang recommended by the WHO (World Health Organization) in 1981. The age grouping covered five years, and the probability of death in the 0 age group was the infant mortality rate [12]. In this study, we used the abbreviated life table. The abbreviated life table was based on cross-sectional data to obtain known mortality rates for all age groups in a given year (or period); it assumes that the generation born at the same time will die sequentially according to the mortality rates of each age group until the death of the last one. Then, the “probability of death”, “number of deaths”, “number of survivors”, and “life expectancy” of this generation in different age groups are calculated, respectively, and thus, the table of age-at-appearance is compiled. The key to the compilation was to calculate the proportion of the cause-of-death number to the total number of deaths. The basic idea is that if a cause of death is eliminated, the person who dies of the cause of death will not die of the cause of death in the future and will live longer. Cause-eliminated life expectancy (CELE) [10] reflects the severity of the impact of a particular cause of death on the health of the population; it can provide a scientific basis for identifying the priority areas for disease prevention and control. Research shows that CVD death is one of the main factors affecting LE in China.

All of the data analyses were performed by Excel 2016 (Microsoft Corporation, Albuquerque, NM, USA) and SPSS software version 22.0 (IBM, Armonk, NY, USA).

## 3. Results

### 3.1. LE of the Chinese Population

In 2019, the LE of Chinese residents was 79.39 years (77.00 years for men, 82.00 years for women). At the same time, the LE of urban residents was estimated at 80.42 years, higher than the figure of 78.86 years for rural residents. LE in the Eastern region was 80.43 years, higher than that in the Central (79.06 years) and Western (78.18 years) regions (data not shown).

### 3.2. Increases in Life Expectancy after Eliminating Deaths from Major Chronic Diseases

**Figure 1:**
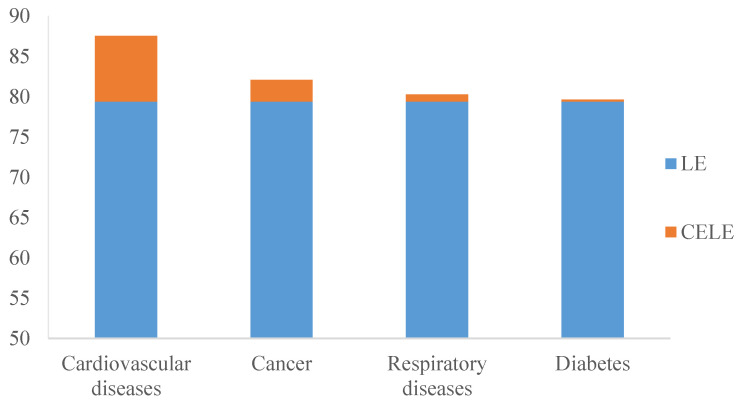
Life expectancy of Chinese residents after eliminating diseases’ deaths, 2019. LE: Life expectancy; CELE: Cause-eliminated life expectancy.

After eliminating the causes of death from four major chronic diseases (cardiovascular disease, cancer, respiratory disease, and diabetes), the LE of Chinese residents increased by 8.13 years, 2.68 years, 0.88 years, and 0.24 years, respectively (Table 1). Among them, we found that the gains in LE were highest after eliminating CVD, followed by cancer, respiratory diseases, and diabetes. The change in LE after the elimination of causes of death from CVD and diabetes was greater in women than in men, and it was greater in men than in women after the elimination of causes of death from cancer and respiratory diseases (Figure 1).

**Figure 2:**
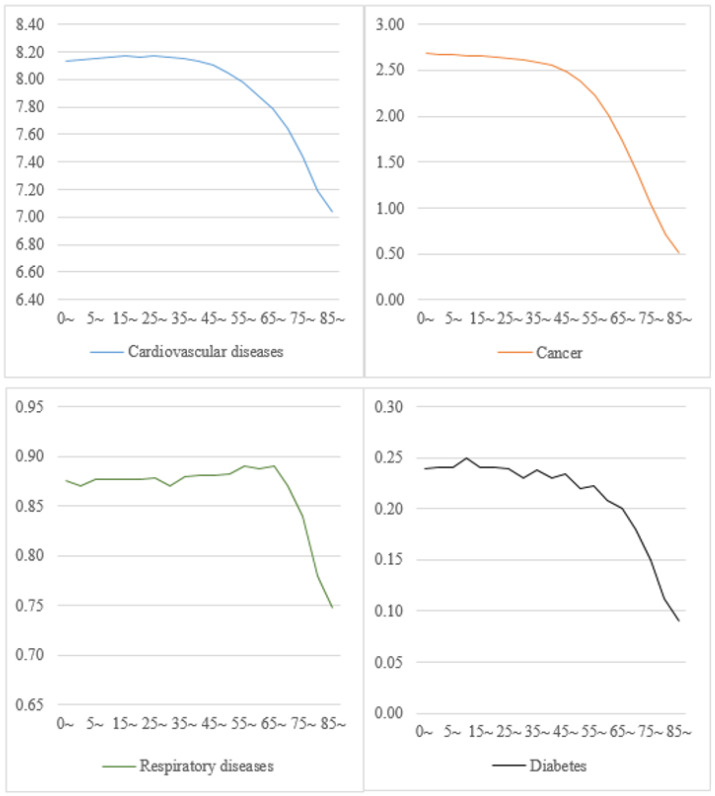
Increases in life expectancy by age group after eliminating diseases’ deaths. China, 2019.

When classified by age, after eliminating CVD deaths, the LE of different age groups increased significantly. Our comparison of different age groups showed that after removing cancer deaths, LE was reduced by 2.68 years for the 0-year-old group and by 0.51 years for the 85+ group. After the elimination of respiratory disease and diabetes as causes of death, LE increased by 0.88 and 0.24 years for the 0-year-old group and by 0.75 and 0.09 years for the 85+ group, respectively (Figure 2).

When classified by region, the LE of residents in the Eastern, Central, and Western regions after removing CVD deaths was 88.07, 88.93, and 85.18 years, representing increases of 7.64, 9.87, and 7.00 years, respectively. LE increased by 2.94, 2.60, and 2.35 years in the eastern, central, and western regions, respectively, after eliminating cancer deaths. After eliminating deaths from respiratory disease, LE increased by 0.67 years in the eastern region, 0.74 years in the central region, and 1.41 years in the western region. LE rose by 0.25 years in the east, 0.23 years in the central part, and 0.24 years in the West after removing deaths from diabetes (Table 2).

### 3.3. Association between Different Categories of Chronic Diseases and Life Expectancy

Furthermore, after eliminating mortality resulting from CVD, cancer, respiratory disease, and diabetes, the LE for the general population increased by 28.26 years. We found that male LE increased more than female LE, while the LE of rural residents increased more than that of their urban counterparts. In addition, eliminating deaths from these causes had a greater effect on the Central region when compared with the Eastern and Western regions. The increase in LE in the Eastern region, however, was higher than in the Central and Western regions after eliminating cancer. (Table 3).

For residents over the age of 65, the impact of the four major types of chronic diseases on LE represents a more significant downward trend. (Figure 2).

The data analysis found that the results were statistically significant. Details are shown in Table 4.

## 4. Discussions

The main findings in the present study indicate that the association of CVD deaths and Chinese residents’ LE was notable. Women suffered a greater loss of LE from CVD disease than men [12,13]. Similarly, the reduction in the LE of rural residents due to CVD was greater than that of urban residents, and it was greater in central areas than in eastern and western areas. Our results are consistent with studies in other countries [14,15]. CVD is the leading cause of death and a major health problem in China. CVD is also the world’s leading cause of death, with an estimated 17.9 million deaths in 2016, accounting for 31 percent of all deaths worldwide [16,17,18]. Most CVD can be prevented by adopting population-oriented strategies that address risk factors such as smoking, unhealthy diet, obesity, physical inactivity, and harmful use of alcohol [19].

In addition, our comparison of different age groups shows that the loss of life years due to cancer-related deaths decreases gradually with increasing age, with greater changes in men’s LE than in women’s, with an increase of 3.15 years for men and 2.07 years for women, compared with 3.60 years for Canada. This was consistent with other studies [20,21,22], which also found greater changes in urban LE than in rural LE and greater changes in the east than in the central and west. Liu [23] conducted research on the impact of leading causes of death on LE in China. His research showed that deaths from cancer tend to occur at younger ages; hence, he thought that the targeted population for cancer prevention and control efforts needed to be expanded. He also believed that early diagnosis was important in cancer prevention and that secondary preventive actions, such as screening programs, should be extended to include younger people in order to achieve a reduction in mortality.

Another finding of our study was that LE increased by 0.88 and 0.24 years, respectively, after removing respiratory disease and diabetes. According to the WHO (https://www.who.int/news-room/fact-sheets/detail/diabetes) (accessed on 15 December 2021), the prevalence of diabetes in the world is of major concern. In 2014, 422 million people (or 8.5 percent of the population) had diabetes, compared with 108 million (4.7 percent) in 1980 [24,25]. The diabetes epidemic has had major health and socio-economic impacts [26,27,28], particularly in developing countries. A favorable healthy environment is therefore needed to address the risk factors for diabetes. To reduce the incidence and impact of diabetes, the population should exercise sensibly and develop healthy eating habits. In addition, the state should provide care and treatment services to help patients with diabetes manage their physical condition.

LE in China rose from 76.34 years in 2015 to 79.39 years in 2019 [29]. According to statistics, in 2019, China’s population aged 65 and above accounted for 13.48% of the total population. According to the standard of an aging society, defined by the World Health Organization as 7% (https://www.who.int/) (accessed on 15 December 2021), China has entered a serious ageing period [30], and the elderly are a group with a high incidence of chronic disease. We should therefore pay more attention to the health of the elderly.

This study has its advantages. Along with adding to the Incomplete Death Registration System in China, the results of this study may be a supplement to other studies based on data from the China Death Surveillance Database. Our research has some limitations that need to be addressed. First, the results in this dataset are a summary of the number of deaths reported at each monitoring point. No adjustment has been made for under-reporting; therefore, the results of the calculation of LE and cause-eliminated life expectancy may be biased. Second, LE only reflects the length of life, not the quality of life. Cause-eliminated life expectancy mainly reflects the influence of different causes of death on LE. Therefore, in order to assess the impact of different causes of death on the quality of life or the health of the population, more comprehensive indicators, such as years of disability (YLDs), disability-adjusted life years (DALY), and healthy life expectancy (HALE), should be used in future studies [31].

## 5. Conclusions

In conclusion, there was a clear association between the four major chronic diseases (cardiovascular disease, cancer, respiratory disease, and diabetes) and the life expectancy of Chinese residents. The impact of mortality from chronic diseases on residents of different genders and regions was also significantly different. We should therefore strengthen the interventions for the control of risk factors such as blood pressure, smoking, and BMI and promote healthy lifestyles in order to reduce the potential loss of life years and to achieve an improvement in life expectancy.

## Figures and Tables

**Table 1:** Increases in life expectancy for different age groups after eliminating diseases’ deaths. China, 2019.

Age Group (Years)	Life Expectancy (Years)	Increase in Life Expectancy (Years)			
		Cardiovascular Diseases	Cancer	Respiratory Diseases	Diabetes
0~	79.39	8.13	2.68	0.88	0.24
1~	78.59	8.14	2.67	0.87	0.24
5~	74.68	8.15	2.67	0.88	0.24
10~	69.74	8.16	2.66	0.88	0.25
15~	64.81	8.17	2.65	0.88	0.24
20~	59.90	8.17	2.64	0.88	0.24
25~	55.00	8.17	2.63	0.88	0.24
30~	50.11	8.16	2.61	0.87	0.23
35~	45.25	8.16	2.59	0.88	0.24
40~	40.44	8.13	2.55	0.88	0.23
45~	35.69	8.10	2.49	0.88	0.23
50~	31.05	8.05	2.38	0.88	0.22
55~	26.53	7.98	2.23	0.89	0.22
60~	22.19	7.89	2.01	0.89	0.21
65~	18.00	7.79	1.73	0.89	0.20
70~	14.12	7.64	1.40	0.87	0.18
75~	10.66	7.44	1.04	0.84	0.15
80~	7.69	7.19	0.71	0.78	0.11
Above 85	5.05	7.04	0.51	0.75	0.09

**Table 2:** Increases in life expectancy by gender and type of residential district after eliminating diseases’ deaths. China, 2019.

Rank	All		Men		Women		Urban Areas		Rural Areas	
	Disease Type	Increase in Life Expectancy	Disease Type	Increase in Life Expectancy	Disease Type	Increase in Life Expectancy	Disease Type	Increase in Life Expectancy	Disease Type	Increase in Life Expectancy
1	Cardiovascular diseases	8.13	Cardiovascular diseases	7.19	Cardiovascular diseases	9.07	Cardiovascular diseases	7.54	Cardiovascular diseases	8.45
2	Cancer	2.68	Cancer	3.15	Cancer	2.07	Cancer	2.77	Cancer	2.63
3	Respiratory diseases	0.88	Respiratory diseases	0.93	Respiratory diseases	0.78	Respiratory diseases	0.76	Respiratory diseases	0.94
4	Diabetes	0.24	Diabetes	0.21	Diabetes	0.27	Diabetes	0.28	Diabetes	0.22

**Table 3:** Increases in life expectancy by region of residence after eliminating diseases’ deaths. China, 2019.

Eastern Region		Central Region		Western Region	
Disease Type	Increase in Life Expectancy	Disease Type	Increase in Life Expectancy	Disease Type	Increase in Life Expectancy
Cardiovascular diseases	7.64	Cardiovascular diseases	9.87	Cardiovascular diseases	7.00
Cancer	2.94	Cancer	2.60	Cancer	2.35
Respiratory diseases	0.67	Respiratory diseases	0.74	Respiratory diseases	1.41
Diabetes	0.25	Diabetes	0.23	Diabetes	0.24

**Table 4:** Significance of the correlation between life expectancy and four major chronic non-communicable diseases.

Disease Type	R Value	*p* Value
Cardiovascular	0.998	*p* < 0.001
Cancer	0.999	*p* <0.001
Respiratory diseases	0.998	*p* < 0.001
Diabetes	0.998	*p* < 0.001

## Data Availability

http://ncncd.chinacdc.cn/jcysj/siyinjcx/syfxbg/202101/t20210118_223798.htm (accessed on 15 December 2021).

## References

[1] Preston S.H., Choi D., Elo I.T., Stokes A. (2018). Effect of Diabetes on Life Expectancy in the United States by Race and Ethnicity. Biodemogr. Soc. Biol..

[2] Luy M., Di Giulio P., Di Lego V., Lazarevic P., Sauerberg M. (2020). Life Expectancy: Frequently Used, but Hardly Understood. Gerontology.

[3] Jayatilleke N., Hayes R.D., Dutta R., Shetty H., Hotopf M., Chang C.K., Stewart R. (2017). Contributions of specific causes of death to lost life expectancy in severe mental illness. Eur. Psychiatry.

[4] Canudas-Romo V., Liu L., Zimmerman L., Ahmed S., Tsui A. (2014). Potential gains in reproductive-aged life expectancy by eliminating maternal mortality: A demographic bonus of achieving MDG 5. PLoS ONE.

[5] Kochanek K.D., Anderson R.N., Arias E. (2015). Leading Causes of Death Contributing to Decrease in Life Expectancy Gap Between Black and White Populations: United States, 1999–2013.

[6] Li B. (2021). Tutorial for Outline of the Healthy China 2030 Plan. People’s Medical Publishing House.

[7] (2020). World Health Statistics 2020: Monitoring health for the SDGs, sustainable development goals, World Health Organization. https://www.who.int/data/gho/whs-2020-visual-summary.

[8] Li Q., Ma S., Bishai D., Hyder A.A. (2017). Potential gains in life expectancy by improving road safety in China. Public Health.

[9] Xia X., Yue W., Chao B., Li M., Cao L., Wang L., Shen Y., Li X. (2019). Prevalence and risk factors of stroke in the elderly in Northern China: Data from the National Stroke Screening Survey. J. Neurol..

[10] RuYing H., WeiWei G., Jing P.A.N. (2014). Influence of major death causes on life expectancy in residents from surveillance points in Guizhou, 2012. Mod. Prev. Med..

[11] Fan J., Li G.Q., Liu J., Wang W., Wang M., Qi Y., Xie W.X., Liu J., Zhao F., Li Y. (2014). Impact of cardiovascular disease deaths on life expectancy in Chinese population. Biomed. Environ. Sci..

[12] Riddell C.A., Morrison K.T., Kaufman J.S., Harper S. (2018). Trends in the contribution of major causes of death to the black-white life expectancy gap by US state. Health Place.

[13] Fei F.R., Zhong J.M., Yu M., Gong W.W., Wang M., Pan J., Wu H.B., Hu R.Y. (2017). Impact of injury-related mortality on life expectancy in Zhejiang, China based on death and population surveillance data. BMC Public Health.

[14] Conti S., Farchi G., Masocco M., Toccaceli V., Vichi M. (1999). The impact of the major causes of death on life expectancy in Italy. Int. J. Epidemiol..

[15] Li G.Q., Fan J., Liu J., Wang W., Wang M., Qi Y., Xie W.X., Liu J., Zhao F., Li Y. (2014). Impact of cerebrovascular disease mortality on life expectancy in China. Biomed. Environ. Sci.

[16] Nakayama H., Minematsu K., Yamaguchi T., Miyamoto S., Isobe M., Komuro I., Yazaki Y. (2020). Approval of Stroke and Cardiovascular Disease Control Act in Japan: Comprehensive nationwide approach for prevention, treatment, and patients’ support. Int. J. Stroke.

[17] Dorresteijn J.A., Kaasenbrood L., Cook N.R., van Kruijsdijk R.C., van der Graaf Y., Visseren F.L., Ridker P.M. (2016). How to translate clinical trial results into gain in healthy life expectancy for individual patients. BMJ.

[18] Jaul E., Barron J. (2017). Age-Related Diseases and Clinical and Public Health Implications for the 85 Years Old and Over Population. Front. Public Health.

[19] Grover S.A., Kaouache M., Rempel P., Joseph L., Dawes M., Lau D.C., Lowensteyn I. (2015). Years of life lost and healthy life-years lost from diabetes and cardiovascular disease in overweight and obese people: A modelling study. Lancet Diabetes Endocrinol..

[20] Yang J., Zhao L., Zhang N., Du Z., Li Y., Li X., Zhao D., Wang J. (2021). Cancer death and potential years of life lost in Feicheng City, China: Trends from 2013 to 2018. Medicine.

[21] Yamada S.I., Kurita H., Tomioka T., Ohta R., Yoshimura N., Nishimaki F., Koyama Y., Kondo E., Kamata T. (2017). Healthy life expectancy of oral squamous cell carcinoma patients aged 75years and older. Oral. Oncol..

[22] Yang G., Hu J., Rao K.Q., Ma J., Rao C., Lopez A.D. (2005). Mortality registration and surveillance in China: History, current situation and challenges. Popul. Health Metr..

[23] Liu P., Li C., Wang Y., Zeng W., Wang H., Wu H., Lu J., Sun M., Li X., Chang F. (2014). The impact of the major causes of death on life expectancy in China: A 60-year longitudinal study. BMC Public Health.

[24] Wan X., Ren H., Ma E., Yang G. (2017). Mortality trends for ischemic heart disease in China: An analysis of 102 continuous disease surveillance points from 1991 to 2009. BMC Public Health.

[25] Li F., Wen S., Tang Q., Zhou Q., Hao Y., Sun C. (2020). Impact of injury-related deaths on life expectancy in China, 2016. Cad. Saude Publica.

[26] Manuel D.G., Schultz S.E., Kopec J.A. (2002). Measuring the health burden of chronic disease and injury using health adjusted life expectancy and the Health Utilities Index. J. Epidemiol. Community Health.

[27] Li Y., Schoufour J., Wang D.D., Dhana K., Pan A., Liu X., Song M., Liu G., Shin H.J., Sun Q. (2020). Healthy lifestyle and life expectancy free of cancer, cardiovascular disease, and type 2 diabetes: Prospective cohort study. BMJ.

[28] Tachkov K., Mitov K., Koleva Y., Mitkova Z., Kamusheva M., Dimitrova M., Petkova V., Savova A., Doneva M., Tcarukciev D. (2020). Life expectancy and survival analysis of patients with diabetes compared to the non diabetic population in Bulgaria. PLoS ONE.

[29] Chen H., Qian Y., Dong Y., Yang Z., Guo L., Liu J., Shen Q., Wang L. (2020). Patterns and changes in life expectancy in China, 1990-2016. PLoS ONE.

[30] Fang E.F., Xie C., Schenkel J.A., Wu C., Long Q., Cui H., Aman Y., Frank J., Liao J., Zou H. (2020). A research agenda for ageing in China in the 21st century (2nd edition): Focusing on basic and translational research, long-term care, policy and social networks. Ageing Res. Rev..

[31] Bushnik T., Tjepkema M., Martel L. (2018). Health-adjusted life expectancy in Canada. Health Rep..

